# Abattoir Factors Influencing the Incidence of Dark Cutting in Australian Grain-Fed Beef

**DOI:** 10.3390/ani11020474

**Published:** 2021-02-10

**Authors:** Cameron C. Steel, Angela. M. Lees, D. Bowler, P. A. Gonzalez-Rivas, G. Tarr, R. D. Warner, F. R. Dunshea, Frances C. Cowley, P. McGilchrist

**Affiliations:** 1 School of Environmental and Rural Science, Faculty of Science, Agriculture, Business and Law, University of New England, Armidale, NSW 2351, Australia; angela.lees@une.edu.au (A.M.L.); fcowley@une.edu.au (F.C.C.); peter.mcgilchrist@une.edu.au (P.M.); 2 Animal Health Data, 177 Bennetts Road, Norman Park, QLD 4170, Australia; desbowler@initmedia.com.au; 3 School of Agriculture and Food, Faculty of Veterinary and Agricultural Sciences, The University of Melbourne, Melbourne, VIC 3010, Australia; paula.gonzalez@unimelb.edu.au (P.A.G.-R.); robyn.warner@unimelb.edu.au (R.D.W.); fdunshea@unimelb.edu.au (F.R.D.); 4 School of Mathematics and Statistics, Faculty of Science, The University of Sydney, Sydney, NSW 2006, Australia; garth.tarr@sydney.edu.au; 5 Faculty of Biological Sciences, The University of Leeds, Leeds LS2 9JT, UK

**Keywords:** high pH, feedlot cattle, processing factors, dark meat, weather

## Abstract

**Simple Summary:**

This study was conducted to generate a greater understanding of the abattoir factors that influence the incidence of dark cutting in Australian grain-fed beef. Elucidation of the factors that are associated with an increased risk of dark cutting will allow for the development of effective management strategies to be implemented to reduce dark cutting in feedlot cattle. This will increase profitability across the supply chain for both producers and abattoirs, whom need to collaborate for the minimization of dark cutting.

**Abstract:**

The aim of this study was to evaluate the effect of carcass traits, lairage time and weather conditions during lairage and abattoir factors that impact the incidence of dark cutting in 142,228 grain-fed carcasses, as defined by Meat Standards Australia (MSA) guidelines. This study was conducted over a 12-month period analysing data from cattle that were supplied from seven feedlots and processed at three abattoirs. Abattoir data indicated that the average incidence of dark cutting within the study was 2.8%. Increased wind speeds (WSs) and rain during lairage at the abattoir was associated with an increased risk of dark cutting, whereas variation in ambient temperature and/or relative humidity did not influence dark cutting. Heavier carcasses with whiter fat, larger hump heights, more rib fat, higher marble scores and lower ossification had lower incidences of dark cutting. The factors abattoir, time in lairage, time to grading and grader within Abattoir had significant effects on the incidence of dark cutting. The results from this study suggest that reducing the time in lairage and increasing the time between slaughter and grading are the two major ways to reduce dark cutting in MSA carcasses.

## 1. Introduction

Dark cutting beef is defined by Meat Standards Australia (MSA) as non-compliant on pH when measured at grading, specifically those carcasses that have a high ultimate pH (pH_u_), i.e., pH_u_ > 5.70. Dark cutting beef is associated with deteriorated meat quality, thus beef producers are generally penalized by abattoirs to compensate for reduced saleable quality product [1,2,3]. Non-compliant MSA beef, based on pH, has been estimated to cost the Australian beef industry approximately $55 million per year [1]. McGilchrist, et al. [4] estimated the cost of non-compliant MSA beef to producers equated to approximately $0.50 (AUD) per kg carcass weight or $7.09 (AUD) for every carcass graded within the MSA system during 2009.

Dark cutting is a complex multifactorial problem that is influenced by nutrition and pre-slaughter factors which induce stress, exercise and increase muscle glycogen utilisation. The condition is generally attributed to low muscle glycogen stores at slaughter [5], which is either a function of insufficient glycogenesis on farm [6] or too much glycogenolysis during the pre-slaughter period due exercise or stress. Muscle glycogen concentration at slaughter has been associated with numerous factors including, but not limited to, nutritional status [4,7,8], water supply and quality [6], animal temperament [9,10], gender and climatic conditions [8].

Grain-fed cattle are typically on a higher plane of nutrition than grass-fed cattle, therefore have greater glycogen stores and consequently typically have a lower incidence of dark cutting [4,11]. Additionally, it has been speculated that grain-fed cattle are more acclimated to various stressors, including trucking, machinery and regular interactions with stockpersons [12,13]. A beef quality audit conducted in 2015 by MSA determined that the incidence of dark cutting in grain-fed cattle ranged between 1.5% and 2.5%, with a peak incidence of 2.5% occurring in March [14]. This variability in the incidence of dark cutting in grain-fed cattle may be associated with various abattoir factors including: (i) climatic conditions during lairage, particularly heat load; (ii) lairage conditions, i.e., transport time, arrival time, time in lairage and (iii) post slaughter management, i.e., time to grading and carcass traits. Furthermore, there may be interactions between carcass traits, time in lairage and climatic conditions during lairage, which may have a cumulative impact on the incidence of dark cutting. This study was undertaken to develop a comprehensive understanding of the factors that influence dark cutting of grain-fed beef in Australia. To meet these objectives the study investigated data from three abattoirs and seven of their suppling feedlots over a 12-month period to identify risk factors associated with dark cutting.

## 2. Materials and Methods

### 2.1. Data Sourced

Data were obtained from three abattoirs and seven feedlots (Table 1). All three abattoirs had a minimum of two suppling feedlots. Additionally, there was one feedlot that supplied to two abattoirs. Abattoirs were defined as Abattoir A to Abattoir C and feedlots were defined as Feedlot A to Feedlot G (Table 1).

### 2.2. Weather Data

Climatic data were obtained from weather stations installed at each abattoir (n = 3) to capture onsite climatic conditions during lairage. Weather stations were located close to lairage pens, without being obstructed by shade and surrounding structures. These data were used to calculate the temperature humidity index (THI), heat load index (HLI) and accumulated heat load (AHL). The HLI was calculated to the reference animal as described by Gaughan, et al. [15], where the reference animal was considered as a clinically healthy black Angus steer < 100 days on feed. The THI was calculated using the following equation;

THI = 0.8 × TA + ⌊ (RH/100) × (TA − 14.4) ⌋ + 46.4

where RH = Relative Humidity (%) and TA = wet bulb or dew point temperature

The HLI was calculated using the equation described by Gaughan et al. [15] where the HLI equation takes the following forms;

(i) A nonlinear regression which applies when BGT is greater than 25 °C
  HLI_BGT>25_ = 8.62 + (0.38 × RH) + (1.55 × BGT) − (0.5 × WS) + [e^2.4−WS^ ]
(ii) A linear model which applies when BGT falls below 25 °C;
  HLI_BGT<25_ = 10.66 + (0.28 × RH) + (1.3 × BGT) − WS
  where RH = Relative Humidity (%); BGT = Black Globe Temperature (°C); WS = wind speed (m/s); and e = the base of the natural logarithm (approximate value of e = 2.71828).

The AHL was calculated using the equations described by Gaughan et al. [15] established the following equations:

(i) If [HLI_ACC_ < HLI_Lower Threshold_, (HLI_ACC_ − HLI_Lower Threshold_)/M]; and
(ii) If [HLI_ACC_ > HLI_Upper Threshold_, (HLI_ACC_ − HLI_Upper Threshold_)/M, 0]
  where HLI_ACC_ = the actual HLI value at a point in time; HLI_Lower Threshold_ = the HLI lower threshold where cattle will dissipate heat (e.g., 77); HLI_Upper Threshold_ = the HLI upper threshold where cattle will gain heat (e.g., 86); and M = number of measures per hour, i.e., number of times HLI data are collected per hour; If every 10 min, then M = 6 (Gaughan et al.) [15].

### 2.3. Meat Standards Australia Carcass data

Carcass feedback data from all producers was sourced from the MSA database for statistical analyses. Carcass data were obtained for the 12-month period, which included a total of 142,228 consigned carcasses. When aggregating the data, the following variables were identified and established within the dataset for each carcass:

Number of animals (n_animal) = number of animals in each lot

Grading date (gradedate) = the date of carcass grading

Carcass identification (bodyno) = the abattoirs body number for each carcass

Grader identification (grader) = the graders identifier

Hormone growth promotant status (HGP) = HGP status (Yes or No)

Carcass sex (sex) = Steers (Male) or heifers (Female)

Carcass measurements were performed by graders accredited with both MSA grading and AUS-MEAT chiller assessment [16]. The carcass measurements include:

Hump height, measured in 5 mm gradients and primarily used as an indicator of tropical breed content [16].

Fat color, determined from the intermuscular fat lateral to the rib eye muscle. It was assessed on the chilled carcass and scored against AUS-MEAT fat color reference standards [17].

Meat color, considered as the predominant color of the rib eye muscle (*longissimus thoracis et lumborum*) as measured on the chilled carcass at the bloomed rib eye muscle face scored against AUS-MEAT color reference standards [17]. Meat color has a scale of 1 to 7, with carcasses in the range of 1B to 3 acceptable for MSA.

MSA Marbling score, a measure of the fat deposited between individual fibers in the rib eye muscle ranging from 100 to 1190 in increments of 10. Marbling is assessed at the quartering site of the chilled carcass and is calculated by evaluating the amount, piece size and distribution of marbling in comparison to the MSA reference standards [16,17].

Rib fat depth (mm), is the depth of subcutaneous fat measured at the quartering site in the chilled carcass approximately 75% of the way along the rib eye muscle [17].

Ossification score, measured following the guidelines from the United States Department of Agriculture [18]. Ossification provides a scale between 100 and 590 in increments of 10 for MSA which is an assessment of physiological age of a bovine carcass. It is a measure of the calcification in the spinous processes in the sacral, lumbar and thoracic vertebrae [17].

Ultimate pH and loin temperature, measured in the rib eye muscle (*longissimus thoracis et lumborum*) of the chilled carcass at the quartering site approximately 8–48 h post-mortem. Loin temperature and pH are measured using an MSA approved pH Meter (TPS MC-80 or TPS WP-80M; TPS Pty Ltd., Springwood, Brisbane, Australia). The pH and temperature probes were inserted into the muscle in close proximity to each other and were allowed to stabilize prior to data collection. The MSA grading standards define that carcass grading cannot commence until loin temperature is below 12 °C [17].

Hot standard carcass weight (HSCW), measured at the end of the slaughter chain in kilograms with Carcass dressed to AUS-MEAT carcass standards [17].

Eye muscle area (EMA), measured using the AUS-MEAT EMA standard grid as the number of square centimeters of longissimus thoracis et lumborum at the quartering site [17], this is not an MSA requirement but is recorded by abattoirs.

### 2.4. Lairage Data

Arrival time: Arrival time of cattle to the abattoir. Taken from photographs or scans of hand written trucking sheets for groups of cattle entering abattoir. These were aligned with MSA feedback using the variables kill date, feedlot and number of cattle. Often the number of cattle on the trucking sheets were incorrect, this was rectified by searching individual animal National Livestock Identification System (NLIS) numbers () from feedlot data and matching to MSA feedback to identify lot numbers, but was extremely time consuming. From these data Time in Lairage (kill time–arrival time) and time off feed (Time in lairage + transport time) were determined.

### 2.5. Statistical Analysis

All data management and analysis was performed in R 3.6.2 software. Data merging and manipulation was performed using the ‘dplyr’ package, whilst exploratory visualizations were generated within the ‘ggplot2′ package and summary tables were generated using the package ‘Table 1′. Time series manipulations were conducted utilizing the ‘tsibble’ package [19,20,21].

Data was merged from various sources as previously described. Where possible, data were merged at an individual animal level via their unique NLIS identification number. For the lairage data, lot level information was merged by kill date, Abattoir and lot size.

The base models, with dark cutting as the dependent variable (pH > 5.7 at grading), were fit as generalized linear models (GLM) with a logistic link function, such that the estimated coefficients may be interpreted as log-odds (or odds ratios when exponentiated). Where appropriate, Abattoir and producer were always included as main effects.

Variables were fitted as GLM with a logistic link function, such that the estimated coefficients may be interpreted as log-odds (or odds ratios when exponentiated). Where appropriate, feedlot and abattoir were always included as main effects. Most variables showed up as statistically significant, but this was largely associated with the sample size.

The model for weather in lairage on day of arrival at the Abattoir was fit using GLM using the ‘lme4′ package. Within the model, sex and HGP status were included as fixed effects and producer and kill date as random effects. The factors included were: Solar radiation (SR) (W/m^2^), wind speed (WS) (m/s), rain (mm), RH (%) and ambient temperature (TA) (°C). Model outputs were visualized and tabularized with the ‘sjPlot’ package. This includes the forest plots for coefficients and tables of estimated odds ratios. Posthoc pairwise difference estimates were found using the ‘emmeans’ package.

## 3. Results

### 3.1. Incidence of Dark Cutting

Abattoir A, B and C had dark cutting incidences of 2.86%, 3.15% and 2.56% over the duration of the study (Table 2). Abattoir was a significant variable in the base model and the odds of carcasses being classified as non-compliant MSA based on pHu at Abattoir B were 3.66 times the odds of dark cutting occurring at Abattoir A (*p* < 0.001; Table 3). There were no differences in likelihood of different odds of dark cuttings occurring at Abattoir C in comparison to Abattoir A (*p* = 0.561).

### 3.2. Lairage Factors Influencing Dark Cutting

Time in lairage was highly variable and was associated with increased incidence of dark cutting (*p* < 0.001, Table 4). The odds of high pH for animals that had an additional hour in lairage were 1.06 times the odds of animals without the additional hour of lairage (Table 4). Additionally, transport time and time in lairage were used to estimate time off feed. Time cattle were off feed was analyzed in a separate model and was also associated with an increased incidence of dark cutting (*p* < 0.001) and had an odds ratio of 1.06 (Table 4).

### 3.3. Climate Conditions during Lairage

During lairage, WS and rainfall influenced the odds of carcasses being classified as pH non-compliant (Table 5). As average WS increased on day 0 by 1m/s, the odds of arriving cattle failing on pH were 1.0288 (*p* < 0.05) times the odds of cattle failing when exposed to WS 1 m/s less. A similar result was shown with maximum and minimum WS, which had an odds ratio of 1.032 and 1.051, respectively. Rain on the day of arrival at the abattoir also had a significant effect on the incidence of dark cutting (*p* < 0.001, Table 5). Rain on day 0 increased the likelihood of cattle failing on pH by 1.23 times the odds of cattle failing exposed to 1 mm total rain less (Table 5). There was no relationship between RH, TA or SR and odds of dark cutting within this model. Temperature humidity index was also not significant in when modelled.

### 3.4. Processor Factors Influencing Dark Cutting

Time to Grading.

Time post mortem before carcass grading influenced the likelihood of MSA dark cutting. Time to grading was categorized into six categories, encompassing 4 h periods between 8 and 24 h, 24 to 48 h and then ≥48 h (Table 6). As time to carcass grading increased beyond time category 1 (8 to 12 h), the odds of a carcass being classified as non-compliant for pH decreased. Time category 2, 4, 5 and 6 were 0.691, 0.698, 0.414 and 0.645 times the odds of time category 1 for non-compliant MSA carcasses based on high pH (*p* < 0.001; Table 3). However, there was no difference in the odds of dark cutting between time category 1 and 3 (*p* = 0.514; Table 3).

### 3.5. Carcass Factors Influencing Dark Cutting

Fat Colour.

Relative to fat color 0, animals with higher fat color score were more likely to have high pH and a higher incidence of dark cutting. Fat color was significant in the model (*p* < 0.001; Table 3) with the odds of fat color groups 1, 2, 3 and 4+ being 1.972, 2.465, 4.076 and 5.362 times more likely to be classified as pH non-compliant than the odds of fat color group 0. This was reflected in the proportion of non-compliant carcasses for each fat color group (Table 7).

#### 3.5.1. Hot Standard Carcass Weight

The mean HSCW was 332 ± 60.5 kg, although it varied among producers (Table 1). The HSCW influenced the incidence of dark cutting, where a 10 kg increase in HSCW was 0.919 times the odds of being a dark cutter compared to a carcass 10 kg lighter (*p* < 0.001). This is further emphasized when carcasses were grouped by sex and compared. The average HSCW was lower when carcasses had a pH > 5.7, irrespective of sex.

#### 3.5.2. Ossification

Ossification was significant in the model (*p* < 0.001; Table 3). As ossification score increased by 10 units, the odds of the carcass being classified as pH non-compliant were 1.055 times higher compared with a carcass with an ossification score 10 units lower.

#### 3.5.3. Marbling

While Feedlot A had the highest mean marble score with 619 ± 227 the average marble scores across producers was 387 ± 140 (Table 1). Marbling was evaluated categorically by group rather than as a continuous absolute score in the base model and had a significant effect on the pH compliance of carcasses. Marbling was significant in the model for the 300 to 500 (*p* < 0.001), 500 to 700 (*p* = 0.001) and > 700 (*p* < 0.001) marbling categories (Table 3). The odds of each marbling group having a high pHu, in ascending marble score, were 0.571 (MSA Marble 300 to 500), 0.685 (MSA Marble 500 to 700) and 0.401 (MSA Marble 700+) times the odds of when compared with the lowest MSA marbling category (MSA Marble 100 to 300). Relative to the low marbling group (100–300), the other marbling groups exhibit lower odds of being classified as non-compliant MSA pH as marbling score increased (Table 3).

#### 3.5.4. Rib Fat

Rib fat was significant in the model (*p* < 0.001) and had a positive influence on compliant pH (Table 3). As rib fat increased by 1 mm, the odds of a carcass being classified as pH non-compliant was 0.844 times the odds of a carcass with a 1 mm lower rib fat. Higher means were seen in the pHu < 5.7 categories for both male and female carcasses. Any score of 30 mm rib fat or more was only captured under the pH pass category for females, and males had no scores over 25 mm with a pH fail.

#### 3.5.5. Hump Height

Hump height was significant in the model (*p* < 0.001; Table 3). As hump height increased by 10 mm the odds of a carcass being classified as pH non-compliant was 0.950 times the odds of a carcass with a hump measuring 10 mm less.

## 4. Discussion

Dark cutting is an economic burden on Australian abattoirs costing the industry around 55 million dollars (AUD) per year in losses due to downgrades in saleable meat quality [1]. This study has highlighted several factors that have a significant effect on the incidence of dark cutting within the processing of grain-fed beef. Due to dark cutting being a complex, multifactorial issue and the relatively low incidence, it is difficult to generate predictive models to determine the factors associated with dark cutting. However the use of odds ratios as utilized within this study can be an informative tool to understand the risk factors. The risks evaluated within this study have defined the factors that are associated with an increased incidence of dark cutting in Australian grain feed beef. The following abattoir factors had a significant effect on carcasses graded non-compliant on pH^u^.

### 4.1. Time in Lairage and Time off Feed

Results from the current study suggest that increasing the amount of time cattle spent in lairage was associated with an increase in dark cutting. This aligns with the work by Jones, et al. [22] showing that increasing fasting from 4 to 24 h increased the pHu of meat in steers. Jones, et al. [23] showed that increases in feed and water withdrawal in cattle from 0 to 48 h led to increased pHu. Increasing the time spent in lairage would also influence the time that cattle are exposed to the abattoir lairage environment and the resultant stress of human activity, novel environments and more confined spaces. Additionally, the period between leaving the feedlot and slaughter comprises a multitude of stressors [24], thus reducing this period will minimize the compounding impacts on muscle glycogen stores and ensure that glycogen concentrations remain as high as possible at slaughter. Time in lairage can be viewed as time in which stress and exercise can occur, reducing muscle glycogen concentration during that period. Hence, reducing the time in lairage is one of the main targets to lower the incidence of dark cutting in grain-fed cattle.

### 4.2. Time to Grading

The time between slaughter and grading is required to allow post mortem glycogenolysis to occur, producing lactate and hydrogen ions, which subsequently cause muscle pH to decline from around neutral at the time of death to an ultimate level around 5.5 [25,26]. A limited pH decline results in a high pHu (pHu > 5.70) and a dark color at grading, as opposed to a full pH decline to 5.4–5.5, which results in a bright cherry-red color at grading [27]. The time to grading results within the current study suggest that some carcasses are being graded before they reach pHu and are falsely classified as pH non-compliant. This suggests that some carcasses are graded too early and that grading should occur after a minimum of 20 h. Processors aiming to grade as early as possible would need to push their chilling regime harder to cool carcasses and this slows the rate of glycogenolysis and can compound the effect with shorter grade times.

### 4.3. Fat Color

The effect of fat color on the incidence of dark cutting in this study is an interesting outcome. The fat color of grass-fed beef typically appears more yellowish than the whiter fat color of grain-fed beef due to the increased consumption levels of carotenoids [28]. The results of this study showed that carcasses with higher or yellower fat color scores, which are predominantly only seen in grass-fed carcasses when graded, were shown to have a higher incidence of dark cutting. This increased yellowing of fat in grain-fed carcasses may be attributed to a state of reduced liver function causing jaundice [29] during or prior to entering the feedlot. If liver function is reduced due to acidosis, fluke or abscess, the underlying illness, coupled with lowered feed intake and slower growth rates, would lower glycogen at slaughter and increase the likelihood of dark cutting [30,31,32]. Even though there were no grass-fed cattle in the dataset, due to the variations in fat color, it is possible that these animals were either short days on feed coming off grass. Although this result was still evident when days on feed (DOF) was included in the statistical model indicating that this finding is not driven by DOF alone. As such, further investigations are required to understand the factors relating to higher, or increasing, fat color scores in grain-fed cattle and its relationship with non-compliant MSA pH. Walker, et al. [33] found that carcasses with extremely yellow fat predominantly occurred in older cattle that had been grass-fed. This could be the result of the accumulation of carotenoids over time in response to seasonal nutritional variations, however the true relationship between fat color, time on grain feed and muscle glycogen has had limited investigations. However, the results from this study suggest that a strong relationship exists.

### 4.4. Climatic Conditions during Lairage

During lairage, there was no relationship between RH, TA or SR and odds of dark cutting. Increasing WS and rainfall increased odds of dark cutting. This may be due to heightened stress response in cattle within lairage during storm activity. Cattle entering a new environment are already in a state of increased stress, rain and wind can reduce visibility and impair hearing, increasing vulnerability in a mob of cattle acclimating to a new environment [34,35,36]. Furthermore, feedlot cattle consigned during periods of rain are generally dirtier and will usually undergo extra washes before slaughter. Washing cattle is a stressful event so increasing the duration of the event will further reduce muscle glycogen and increase the likelihood of high pHu [37]. Within this study, it is not yet apparent if the relationship between dark cutting and climatic conditions is associated with seasonal variability. Until knowledge regarding these factors is developed, reducing time in lairage, as highlighted within this study, may reduce the incidence of dark cutting.

### 4.5. Hot Standard Carcass Weight, Marbling and Rib Fat

Findings from this study show that heavier carcasses with higher MSA marbling scores and greater rib fat depths have lower incidences of dark cutting. These characteristics all pertain to an animal’s mode of nutrition and metabolisable energy intake in the finishing phase and therefore complement the results relating to nutrition. Animals finished on higher energy diets produce fatter carcasses in all depots, including intramuscular and subcutaneous depots or marbling and rib fat [38]. Furthermore it can be assumed that heavier carcasses at the same age as lighter carcasses are also the result of having better nutrition levels [4]. This is in alignment with the findings of Kreikemeier, et al. [39] who found a decrease in DFD incidence of 0.94% to 0.6% as the mean carcass weight of a slaughter group increased. Findings of McGilchrist et al. [4] support these results for rib fat depth and carcass weight. Furthermore, in carcasses with fat thicknesses below 7.6 mm, muscle pHu values were higher and muscle color appeared darker [40].

### 4.6. Ossification

Groups of animals with a higher average age and maturity compared to other groups have a higher incidence of dark cutting. This result aligns with the findings of McGilchrist et al. [4] who found that cattle that grow faster, as indicated by having lower ossification scores compared to other cattle of the same carcass weight, have lower incidences of non-compliant pH. Good nutrition increases animal growth rates and increases muscle glycogen concentrations [41] which decreases the occurrence of non-compliant pH. Another possible reason for this finding could be the fact that younger cattle indicated by lower ossification scores have a higher proportion of fast glycolytic type IIX muscle fibers than older cattle [42]. A higher proportion of these muscle fibers enhances their ability to synthesize muscle glycogen [4]. Therefore, it is likely that younger cattle will have higher muscle glycogen content. Wegner, et al. [43] also supports this concept as they demonstrated that paler colored meat was related to cattle having a higher frequency of type IIX muscle fibers. Evidently, in order to minimize the cost of dark cutting, producers should sell cattle at younger ages or ensure that if older cattle are sold for slaughter they are appropriately managed in terms of nutrition in the weeks prior to slaughter so as to minimize the risk of failing on pH.

### 4.7. Hump Height

Within the current study, as the *Bos indicus* content of cattle increased, as measured by increased hump height, the incidence of dark cutting declined. This finding is supported by Lorenzen, et al. [44] who reported that the incidence of dark cutting was 4.7% and 4.4% for *Bos taurus* and *Bos indicus* carcasses respectively. The decreased incidence of dark cutting in *Bos indicus* cattle could be attributed to their ability of being less susceptible to stress than *Bos taurus* cattle as shown by Tyler, et al. [45]. However, some studies have found no muscle color differences between *Bos indicus* type carcasses and *Bos taurus* [34,40]. This could be the result of relatively small numbers of cattle analyzed and low proportions of *Bos indicus* cattle compared to *Bos taurus*. It is reasonable to conclude that *Bos indicus* cattle have lower incidences of dark cutting than *Bos taurus* due to their decreased susceptibility to stress and therefore a lower level of muscle glycogen depletion during the pre-slaughter period.

## 5. Conclusions

The current study has highlighted that a longer period between slaughter and carcass grading was associated with a decreased incidence of dark cutting, with carcasses graded during the 24–48 h post mortem window having the lowest odds of non-compliant pH and meat color. Conducting carcass grading after carcasses reach pHu reduces the likelihood of false positive pH non-compliance and minimizes income losses across the value chain. Currently, AUSMEAT guidelines state that carcasses can be graded from 8 h post-mortem if electrically stimulated. However, this study has shown that carcass grading at 8 h post-mortem does not allow all carcases to reach pHu. All three abattoirs in this study graded carcasses from 9 h indicating that a re-grading strategy may be viable for re-evaluating carcasses that have not reached pHu when initially graded at 9 h. Abattoirs should also attempt to reduce the time cattle spend in lairage as an increased lairage duration was associated with an increased incidence of dark cutting. Dark cutting in grain-fed beef remains a multifactorial issue, this study has identified that there are opportunities for abattoirs to reduce the incidence of dark cutting in these cattle.

## Figures and Tables

**Table 1:** Number of carcasses with attributes across producers and three processing facilities.

Feedlot	A (n = 7472)	B (n = 18,546)	C (n = 18,989)	D (n = 62,349)	E (n = 6082)	F (n = 8237)	G (n = 19,147)	Overall (n = 140,822)
ABATTOIR								
A	0 (0%)	0 (0%)	0 (0%)	62,349 (100%)	6082 (100%)	0 (0%)	0 (0%)	68,431 (48.6%)
B	0 (0%)	18,546 (100%)	4000 (21.1%)	0 (0%)	0 (0%)	0 (0%)	19,147 (100%)	41,693 (29.6%)
C	7472 (100%)	0 (0%)	14,989 (78.9%)	0 (0%)	0 (0%)	8237 (100%)	0 (0%)	30,698 (21.8%)
HGP								
Y	7472 (100%)	0 (0%)	15,899 (83.7%)	55,129 (88.4%)	5741 (94.4%)	8237 (100%)	9933 (51.9%)	102,411 (72.7%)
N	0 (0%)	18,546 (100%)	3090 (16.3%)	7220 (11.6%)	341 (5.6%)	0 (0%)	9214 (48.1%)	38,411 (27.3%)
SEX								
F	7221 (96.6%)	2488 (13.4%)	14,166 (74.6%)	10,105 (16.2%)	6 (0.1%)	4756 (57.7%)	1401 (7.3%)	40,143 (28.5%)
M	251 (3.4%)	16,058 (86.6%)	4823 (25.4%)	52,244 (83.8%)	6076 (99.9%)	3481 (42.3%)	17,746 (92.7%)	100,679 (71.5%)
Hot standard carcass weight (kg)								
Mean (SD)	287 (29.5)	424 (43.2)	298 (57.2)	324 (35.3)	333 (37.5)	260 (24.4)	349 (62.6)	332 (60.5)
Median [Min, Max]	288 [197, 381]	426 [196, 694]	281 [169, 554]	324 [102, 483]	332 [187, 471]	259 [172, 348]	351 [61.0, 602]	324 [61.0, 694]
OSSIFICATION (100–590)								
Mean (SD)	159 (28.6)	168 (55.5)	154 (23.4)	153 (18.4)	151 (13.2)	143 (16.9)	163 (37.4)	156 (30.4)
Median [Min, Max]	150 [110, 400]	160 [100, 590]	150 [100, 400]	150 [100, 500]	150 [100, 250]	140 [100, 280]	160 [100, 590]	150 [100, 590]
MSA MARBLING (100–1190)								
Mean (SD)	380 (56.1)	619 (227)	392 (72.5)	351 (54.5)	353 (48.8)	358 (52.6)	301 (113)	387 (140)
Median [Min, Max]	360 [210, 590]	560 [100, 1190]	360 [120, 980]	350 [100, 1000]	350 [100, 780]	350 [180, 600]	300 [100, 1050]	350 [100, 1190]
RIB FAT (mm)								
Mean (SD)	7.43 (2.04)	11.5 (4.50)	7.59 (2.48)	9.08 (3.41)	9.92 (3.71)	6.22 (1.49)	8.74 (3.58)	8.93 (3.61)
Median [Min, Max]	7.00 [1.00, 25.0]	10.0 [3.00, 55.0]	7.00 [2.00, 56.0]	8.00 [1.00, 60.0]	9.00 [1.00, 32.0]	6.00 [2.00, 40.0]	9.00 [1.00, 55.0]	8.00 [1.00, 60.0]
HUMP HEIGHT (mm)								
Mean (SD)	49.4 (9.18)	66.3 (18.1)	51.0 (12.2)	64.0 (17.3)	66.0 (14.9)	47.5 (9.08)	67.1 (19.9)	61.3 (17.7)
Median [Min, Max]	45.0 [20.0, 150]	65.0 [15.0, 160]	45.0 [20.0, 140]	60.0 [15.0, 265]	65.0 [30.0, 220]	45.0 [20.0, 170]	65.0 [20.0, 280]	60.0 [15.0, 280]
DAYS ON FEED (days)								
Mean (SD)	82.2 (17.0)	285 (92.0)	96.3 (36.3)	105 (25.3)	98.2 (7.07)	61.0 (3.05)	136 (38.2)	128 (76.3)
Median [Min, Max]	82.0 [8.00, 279]	223 [22.0, 565]	83.0 [43.0, 256]	103 [8.00, 282]	100 [70.0, 100]	60.0 [60.0, 70.0]	134 [69.0, 440]	103 [8.00, 565]

**Table 2:** Number of carcasses classified as compliant (pH ≤ 5.69) and non-compliant (pH ≥ 5.70) across the three processing facilities.

Abattoir	Total Carcasses	Compliant	Non-Compliant	Proportion Non-Compliant
A	68 431	66 474	1 957	2.86%
B	41 693	40 379	1 314	3.15%
C	30 698	29 913	785	2.56%

**Table 3:** Base model for pH fail odds ratio prediction including significant variables.

Predictors	Odds Ratio	Confidence Interval	Significance
Intercept	0.197	0.131–0.294	*p* < 0.001
DOF (10 day increments)	1.019	1.009–1.030	*p* < 0.001
HCSW (10 kg increments)	0.919	0.912–0.927	*p* < 0.001
HGP Status	2.292	2.034–2.584	*p* < 0.001
Fat Color (1)	1.972	1.772–2.195	*p* < 0.001
Fat Color (2)	2.465	2.204–2.756	*p* < 0.001
Fat Color (3)	4.076	3.357–4.949	*p* < 0.001
Fat Color (4+)	5.362	3.752–7.663	*p* < 0.001
Time to Grading (12–16 h)	0.691	0.608–0.785	*p* < 0.001
Time to Grading (16–20 h)	0.949	0.813–1.108	*p* = 0.514
Time to Grading (20–24 h)	0.698	0.582–0.837	*p* < 0.001
Time to Grading (24–48 h)	0.414	0.311–0.552	*p* < 0.001
Time to Grading (48 h +)	0.645	0.549–0.757	*p* < 0.001
Hump Height (10 mm increments)	0.950	0.931–0.970	*p* < 0.001
Rib Fat	0.844	0.833–0.855	*p* < 0.001
MSA Marble (300–500)	0.571	0.521–0.625	*p* < 0.001
MSA Marble (500–700)	0.685	0.551–0.852	*p* = 0.001
MSA Marble (700+)	0.401	0.282–0.568	*p* < 0.001
Ossification (10 score increments)	1.054	1.046–1.063	*p* < 0.001
Sex (Steer)	1.145	1.036–1.267	*p* = 0.008
Abattoir B	3.659	2.491–5.374	*p* < 0.001
Abattoir C	0.880	0.572–1.353	*p* = 0.561
Feedlot B	1.266	0.876–1.829	*p* = 0.208
Feedlot C	0.950	0.682–1.322	*p* = 0.762
Feedlot D	2.207	1.729–2.816	*p* < 0.001
Feedlot F	0.512	0.345–0.759	*p* = 0.001
Feedlot G	0.975	0.744–1.276	*p* = 0.855

**Table 4:** The effect of transport, lairage and time off feed on the odds of dark cutting for grain-fed cattle.

Predictors	Base Model		Lairage Model		Transport Model		Time off Feed Model	
	Odds Ratio	Significance	Odds Ratio	Significance	Odds Ratio	Significance	Odds Ratio	Significance
Intercept	0.10	*p* = 0.087	0.03	*p* = 0.015	0.17	*p* = 0.226	0.02	*p* = 0.010
lairage time			1.06	<0.001				
transport time					1.00	0.927		
time off feed							1.06	<0.001

**Table 5:** The odds ratios for the effect of temperature (mean, range, max and min), solar radiation, wind speed, relative humidity and rain during lairage on the incidence of dark cutting.

Predictors	Mean Model		Range Model		Max Model		Min Model	
	Odds Ratio	Significance	Odds Ratio	Significance	Odds Ratio	Significance	Odds Ratio	Significance
Intercept	0.0116	*p* < 0.001	0.0094	*p* < 0.001	0.0108	*p* < 0.001	0.0091	*p* < 0.001
SR_MEAN Watts/m_^2^	0.9997	*p* = 0.748	0.9996	*p* = 0.576			0.9998	*p* = 0.816
SR_MAX Watts/m_^2^					0.9996	*p* = 0.084		
WS_MEAN (m/s)_	1.0288	*p* = 0.014	1.0268	*p* = 0.034				
WS_MAX (m/s)_					1.0324	*p* < 0.001		
WS_MIN (m/s)_							1.0513	*p* = 0.009
RH_MEAN_	0.9977	*p* = 0.571						
RH_RANGE_			0.9940	*p* = 0.125				
RH_MAX_					0.9963	*p* = 0.362		
RH_MIN_							1.0014	*p* = 0.681
T_A_,_MEAN °C_	0.9897	*p* = 0.336						
T_A, RANGE °C_			1.0154	*p* = 0.183				
T_A, MAX °C_					1.0044	*p* = 0.640		
T_A, MIN °C_							0.9971	*p* = 0.742
Rain (mm)	1.2315	*p* < 0.001	1.2213	*p* < 0.001	1.2141	*p* < 0.001	1.2266	*p* < 0.001

**Table 6:** Time to carcass grading categories showing total carcasses and proportion carcasses graded within each time category.

Time Category	Time to Grading, h	Total Carcasses	Proportion Graded
1 h	8 h to 12 h	8 179	5.81
2 h	12 h to 16 h	63 498	45.09
3 h	16 h to 20 h	19 635	13.94
4 h	20 h to 24 h	26 969	19.15
5 h	24 h to 48 h	3 456	2.45
6 h	≥48 h	19 085	13.55

**Table 7:** Total number of carcasses and percentage non-compliant (pH > 5.70) for each fat color group.

Fat Colour	Total Carcasses	Compliant	Non-Compliant	Proportion Non-Compliant
0	37 244	36 776	468	1.30%
1	67 644	65 410	2 234	3.30%
2	33 261	32 122	1 139	3.40%
3	2 296	2 130	166	7.20%
4 +	377	328	49	13.00%

## Data Availability

The data presented in this study are available on request from the corresponding author. The data are not publicly available due to the data being owned by industry stakeholders.
